# Occurrence of Aflatoxins in Commercial Cereal-based Baby Foods in Iran: A Probabilistic Risk Assessment to Health

**DOI:** 10.22037/ijpr.2021.114631.14961

**Published:** 2021

**Authors:** Moein Bashiry, Hassan Yazdanpanah, Ehsan Sadeghi, Sajad shokri, Leila Mirmoghtadaie, Amir Mohammad Mortazavian, Abdorreza Mohammadi, Amene Nematollahi, Ehsan Hejazi, Hedayat Hosseini

**Affiliations:** a *Department of Food Science and Technology, Faculty of Nutrition Science, Food Science and Technology/National Nutrition and Food Technology Research Institute, Shahid Beheshti University of Medical Sciences, Tehran, Iran. *; b *Food Safety Research Center, Shahid Beheshti University of Medical Sciences, Tehran, Iran. *; c *Department of Toxicology and Pharmacology, School of Pharmacy, Shahid Beheshti University of Medical Sciences, Tehran, Iran. *; d *Department of Food Science and Technology, School of Nutrition Science and Food Technology, Kermanshah University of Medical Sciences, Kermanshah, Iran. *; e *Department of Food Hygiene and Control, School of Veterinary Medicine, Shiraz University, Shiraz, Iran. *; f *Department of Food Safety and Hygiene, School of Health, Fasa University of Medical Sciences, Fasa, Iran. *; g *Department of Clinical Nutrition and Dietetics, Faculty of Nutrition Sciences and Food Technology, National Nutrition and Food Technology, Research Institute, Shahid Beheshti University of Medical Sciences, Tehran, Iran.*

**Keywords:** Aflatoxins, Exposure assessment, Risk ranking, Cancer potency, Monte Carlo simulation

## Abstract

The aim of this study was to assess the occurrence of Aflatoxins** (**AFs) including B_1_, B_2_, G_1_ and G_2_ in commercial cereal-based baby foods by HPLC-FLD method in Iran and related risk assessment in three baby age groups (6-12, 12-18, and 18-24 months) using Monte Carlo simulation approach. Results showed an occurrence ranging from 20% to 60% for B1, B2, and G2 aflatoxins, while AFG1 was not detected in any assessed samples. Exposure and risk assessment was estimated to be two groups (aflatoxin B_1_ and total aflatoxins). The highest estimated dietary exposure to both AFB_1_ and total AFs was estimated for 6-12 months aged babies, representing 5.81 ng/kg BW/day and 8.55 ng/kg BW/day, respectively. Overall, the margin of exposures to AFB_1_ and total AFs were lower than 10,000 in all age groups, indicating a health concern about AFB_1_ and total AFs exposure through cereal-based baby food consumption. High cancer risk for high consumers (P95) of baby food was also estimated in all age groups, calling for immediate intervention due to serious claims that AFB_1_, is a highly carcinogenic component, causes hepatocellular carcinoma. Risk ranking results indicated the presence of AFB_1_ is classified as high risk for babies who consume cereal-based foods, which demands the attention of risk managers to reduce or eliminate this risk for the most vulnerable sector of society, whose aged <24 months.

## Introduction

Mycotoxins are a group of thermally stable low molecular weight (0.3–0.7 kDa) toxic compounds produced by a wide variety of fungal species as their secondary metabolites with a high bioaccumulation ability (1, 2). According to literature, aflatoxins (AFs) are noted as the most studied mycotoxins, among more than 300 identified fungal secondary compounds (3), including B_1_, B_2_, G_1_, and G_2_. AFs produced primarily by *Aspergillus flavus* (produces aflatoxins B_1_ and B_2_) and *A. parasiticus* (produces all B_1_, B_2_, G_1_ and G_2_) (4). Food contaminated by AFs may cause several health problems in consumers, including acute or chronic diseases with mutagenic, carcinogenic, and teratogenic effects (5); hence, AFB_1_, as the most acute hepatocarcinogenic aflatoxin, is classified in group 1: “carcinogenic to humans” by the International Agency for Research on Cancer (IARC) (6). Additionally, based on a report by Raiola A, Tenore GC, Manyes L, Meca G and Ritieni A (7), children exposed to AFs are associated with reduced immunization efficiency, consequently increased susceptibility to infections as well as stunted growth.

A variety of food products (agricultural commodities) can be contaminated with AFs due to either infected grains by fungi or post-harvest contamination (8). Cereal and cereal-based products contaminated with AFs are a global health concern as they are used worldwide to supplement foods in babies’ diets, providing them with energy and nutrients when breast milk is no longer enough to meet their nutritional needs (9). Daily consumption of AFs contaminated cereal-based baby food increases the associated risks to babies’ health, such as stunted growth and deficiencies in warding off infectious diseases. (10). Furthermore, incomplete enzymatic systems in children and babies to eliminate hazardous compounds (11, 12) and their higher sensitivity to toxicogenic compounds make them more vulnerable to AFs and leave them at risk of developing chronic diseases later in life (13). Accordingly, due to extreme health concerns regarding contaminated babies’ diet due to AFs, numerous national and international governmental authorities on public health have established maximum regulatory levels for AFB_1_ i.e., the maximum level established by European Union is 0.10 ng/g in Commission Regulation (EC) No 1881/2006 (14) and by National Standard Organization of Iran is 1.00 ng/g for food products without milk and 0.5 ng/g with milk in infant and baby foods (15).

As the elimination of AFs from food products is extremely difficult, babies are exposed to a high risk of consuming AFs contaminated foods. Therefore, continuous monitoring and dietary risk assessment of AFs in baby food products are necessary to provide critical information for food regulators in setting guideline values for AFs in food products. The risk assessment process is based on a series of steps, including hazard identification, hazard characterization; exposure evaluation; and risk characterization. This approach is one of the best practices when applied to comply with new regulations set by international organizations or developed regions (16). The results from a risk assessment can enable risk managers to effectively establish mitigation strategies or contingency plans for accepting any risks. In terms of evaluating exposure to aflatoxins, there are three different methods that can be considered, namely: point estimate (or deterministic) procedure; the semi-probabilistic method; and the probabilistic model, also known as the Monte Carlo simulation (MCS) (17). The latter method is the most promising due to its capability in identifying uncertainties and variabilities in the long-term exposure estimations as well as in performing risk predictions that are used extensively in food sciences (18). Therefore, in this study, firstly, the AFs occurrence in different cereal-based baby food samples from different commercial brands from Iran market in two cold and warm seasons were analyzed and then dietary exposure to AFs via cereal-based baby foods consumption and consequently risk characterization was estimated by MCS in three different age groups including babies aged 6-12, 12-18, and 18-24 months.

## Experimental

*Chemicals and standards*

AFs standards (B_1_, B_2_, G_1_, G_2_), methanol, acetonitrile, ultrapure water, potassium dihydrogen phosphate, potassium chloride, anhydrous disodium hydrogen phosphate, sodium chloride, nitric acid and potassium bromide, all HPLC-grade with high purity, were purchased from Merck (Darmstadt, Germany). Standard solutions of AFB_1_ and AFG_1_ at 1000 ng/mL and AFB_2_ and AFG_2_ at 200 ng/mL concentrations were prepared weekly and used in order to obtain calibration curves.

*Samples collection*

One hundred and twenty cereal-based commercially available baby food samples from six different manufacturers were purchased from retail outlets in Iran market. According to their production date, samples were classified into two categories: cold season (winter) collected in February 2020 and warm season (summer) collected in August 2020 according to their production date. Forty samples from six brands were prepared so that each sample was a mixture of three separately purchased samples of the same brand collected from different batches during a month (19). The collected samples were kept at 4 ºC until further analysis. However, based on the products’ labels, all samples were suitable for infant consumption from six months after birth, and their main ingredients mostly included flour of wheat, rice, maize, oat and rye, although, in 6 of them, almond was the main ingredient. Most of the purchased samples were originally in powder form (n = 32) and the rest in semi-solid-to semi-liquid (n = 8).

*AFs determination*

*AFs extractions*

The extractions were carried out based on Iranian Standard No.6872 with a slight modification (20). Briefly, 20 g of each sample were homogenized and grounded using a blender (Waring, The USA) for 5 min at 18000 rpm. Then, the homogenized samples were well mixed with 100 mL of 80% methanol in water (v/v) in a conical flask for 5 min more at 18000 rpm in order to extract aflatoxins from the samples’ matrix and filtered through a no. 5 Whatman filter paper. Twenty milliliters of each filtrate were then diluted to a final volume of 100 mL using phosphate-buffered saline (PBS) (pH=7.4) in a microwave glass container and shook vigorously. A 110 mL of the obtained solution was filtered using a filter paper (Whatman no. 5) and slowly loaded in an immunoaffinity column (Libios, France) where aflatoxins can trap as antigen inside the column. The column was then washed with 2 mL acetonitrile and dried under air, afterward eluted with 0.5 mL of 60% methanol in water (v/v), and the extracted AFs were collected and kept at 4 °C until further analysis.

*AFs analysis*

The amounts of AFs were determined using HPL-FLD according to a method established by the Institute of Standard and Industrial Research of Iran (ISIRI; Iranian Standard No. 6872) with some modification (20). The HPLC system consisted of liquid chromatography (Waters e2695, separation module, USA), equipped with a Fluorescence detector (model 2475) that was set 365 nm as the excitation wavelength and 435 nm as the emission wavelength and a quaternary, low-pressure mixing pump and inline vacuum degassing. The sensitivity of the instrument was set on 500 EUFS. Chromatographic separation was performed in an Octadecyl-silica (ODS) column (150 ×4.6 mm I.D., 3 µm) (GL Science) with a column oven which kept the column temperature constant at 50 °C throughout the AFs separation. An isocratic elution mobile phase of acetonitrile, methanol, and HPLC-grade water (2:3:5, v/v) containing 35 μL nitric acid and 12 mg KBr (to derivatization in Kobra cell (Farlib, France)) was used with a flow rate of 1.8 mL/min for a sample volume of 75 µL and a separation time of 10 min. The levels of AFs were determined by comparison of retention times and area values with corresponding standards, and the results were expressed as part per billion (ppb).

*Method validation*

The performance of the used method in the current study for the determination of AFs was evaluated in terms of linearity, the limit of detection (LOD), the limit of quantification (LOQ), recovery, and precision. LODs and LOQs were determined based on a signal-to-noise ratio (S/N) of 3 and 10, respectively, where samples were spiked with different concentrations of AFs. The linearity was determined by injecting different concentrations of AFB_1_ and AFG_1_ at 0.02, 0.06, 0.1, 0.14, 0.18, 0.28, 0.36, 0.64, 0.88, and 1 ng/mL and also AFB_2_ and AFG_2_ at 0.004, 0.012, 0.020, 0.028, 0.036, 0.056, 0.072, 0.110, 0.150, and 0.200 ng/mL in duplicate. The percent of recovery also was calculated by comparing 0.5 ng/mL of mixed AFs standards to baby food samples with the measured concentrations after three times analysis. Within-day (run-to-run) precision of the HPLC-FLD method, expressed as RSDs%, was calculated by extracting and analyzing AFs in one sample seven times at the same condition (21).

*Dietary exposure estimation*

Dietary exposure to AFs categorized into two groups (AFB_1_ and total AFs(sum of AFB_1_, AFB_2_, AFG_1_, and AFG_2_)) were estimated for three different age groups, including babies aged 6-12 months, 12-18 months, and 18-24 months in the Iran population using the following formula (22):

$\mathrm{EDI}=\frac{C\times F}{\mathrm{Body weight}}$

Equation 1.

where EDI is estimated dietary intake (ng/kg BW/day), C is the amount of different AFs levels, and F is the consumption rate of cereal-based baby food assumed to be 100 g/day based on recommended serving size for each brand by producer and using scientific literature reports (23). It should be noted that the body weight ranges for each considered group (6-12 months, 12-18 months, and 18-24 months) assumed to be 7.5-9.6, 9.2-10.9 and 10.6-11.9, respectively, according to WHO standards (24). The C, F, and Body weight followed log-normal, triangle and uniform distributions, respectively, which were used then for probabilistic analysis (Monte Carlo simulation approach) of the estimation of dietary exposure using Crystal ball software (version 11.1.2.3 Oracle) (17). For each age group and both AFB_1_ and total AFs, the Monte Carlo simulation was repeated 100,000 times in order to achieve a dietary intake as accurately as possible and also forecasted 5, 50 and 95 percentiles of the consumer population. 

*Health Risk Assessment*

*The Margin of Exposure (MoE)*

In general, benchmark dose lower confidence limit 10% (BMDL_10_), which is an estimation of the lowest dose that is 95% certain to cause no more than 10% cancer incidence, is suggested to obtain MoE. However, the MoE was estimated by dividing the BMDL_10_ reference value of 400 ng/kg BW/day (25) to 5, 50, and 95 percentiles exposures of AFB_1_ and total AFs, using the Monte Carlo simulation approach. The MoE values, based on the BMDL_10_ from an animal study, equal or higher than 10,000 are considered to be a low public health concern (26).

$\mathrm{MoE}=\frac{\mathrm{BMDL}10}{\mathrm{Exposure}}$

**Equation 2.**

*Cancer Risk*

According to an assessment by EFSA (CONTAM Panel), the cancer potency for both AFB_1 _and total aflatoxins is counted to be equal. It reported exposure to aflatoxins to induce 0.01 and 0.3 additional cancer cases per 100,000 for HBsAg− and HBsAg+ populations, respectively (25). Taking this into consideration and according to the prevalence of HBsAg+ in Iran (1.7%) (27), the cancer risk due to AFB_1_ and total AFs exposures-induced hepatocellular carcinoma was obtained for 5, 50, and 95 percentiles. Therefore, to determine cancer probability, the percentage of the population for both carrier (Pop.HBsAg+% = 0.017) and non-carriers (Pop.HBsAg-% = 0.983) of hepatitis B virus infection in Iran and the carcinogenic potency reported by EFSA (PHBsAg- and PHBsAg+ = 0.01 and 0.3 respectively) put into following formula:

Pcancer = (PHBsAg+ × PopHBsAg +%) + (PHBsAg- × PopHBsAg -%)

*Statistical analysis*

SPSS software v. 26 and Crystal ball software (version 11.1.2.3 Oracle) were applied in this study. As one sample K-S test showed the obtained data were not normal; therefore, a nonparametric test was used (Mann-Whitney) to compare samples from different seasons in terms of aflatoxins contamination (*p* < 0.05). Moreover, Crystal ball software was used to risk assessment values calculations.

## Results and Discussion

*Method performance and Validation*

**Figure 1:**
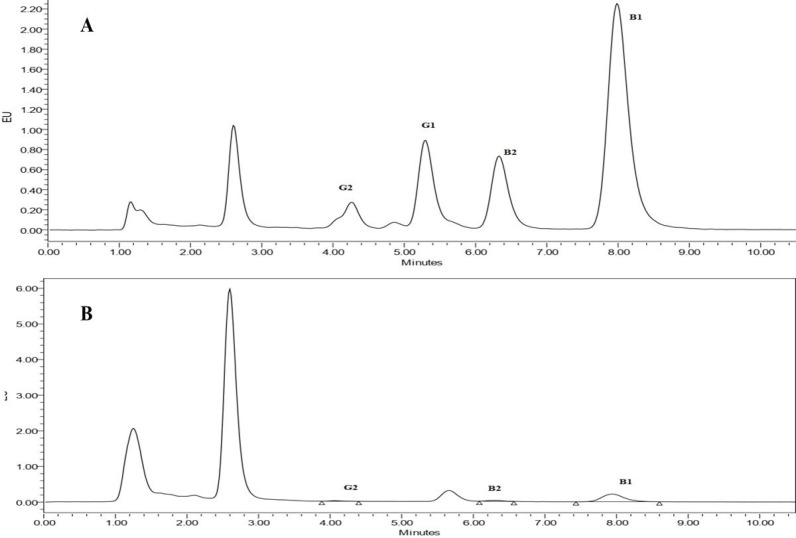
The chromatogram of cereal-based baby food sample obtained by HPLC-FLD. (A) spiked with 0.5 ng/mL aflatoxins B_1_ and G_1_ and 0.1 ng/mL aflatoxins B_2_ and G_2_ and (B) non-spiked samples

In risk assessment studies, reliable analytical methods for risk measurements are required since these data are used to calculate risk factors, which allow risk managers to effectively establish mitigation strategies or contingency plans for accepting any risks. Accordingly, it is internationally authenticated that an appropriate method must be used to ensure precise results. Thus, method validation has received great attention from industrial committees and regulatory agencies (28). For that reason, the performance of the HPLC-FLD method, the used method in the current study, was evaluated based on recovery percentage, the limit of detection (LOD), the limit of quantification (LOQ), coefficient of determination (R^2^), repeatability (RSD), and linear range. The levels of assessed AFB_1_ and AFG_1_ in the range of 0.02-1 ng/mL and the levels of AFB_2_ and AFG_2_ in the range of 0.004-0.2 ng/mL were linear with an R^2^ greater than 0.99 for all AFs. The RSDs for B_1_, B_2_, G_1_, and G_2_ aflatoxins were 7.2%, 7.1%, 6.3%, and 6.3%, respectively, calculated based on comparative peak areas of seven replicates of each sample, indicating good repeatability of the method used. The method also showed a high percentage of analytes recovery for B_1_, B_2_, and G_2_ aflatoxins with percentages of 92%, 105%, and 99%, respectively, while the recovery percentage for G_1_ was 70%. Considering a chromatographic signal to noise ratio of 3 for LOD and 10 for LOQ, the LODs of the method for B_1_, B_2_, G_1_, and G_2_ aflatoxins were 0.013 ng/g, 0.003 ng/g, 0.016 ng/g, 0.003 ng/g, respectively, and LOQs for B_1_, B_2_, G_1_, and G_2_ aflatoxins was 0.04 ng/g, 0.01 ng/g, 0.05 ng/g, 0.01 ng/g, respectively. These results taken together, confirm the validity and performance of the method used, which is compliant with European Union guidelines, as repeatability in terms of RSD% was lower than 20% and the recovery range was between 70–120% (SANTE/11813/2017. Guidance) (29). Comparing the obtained results to the performance criteria set out in the Commission Regulation (EC) No 401/2006 indicates that the method satisfies these performance criteria in terms of repeatability, recovery, and reproducibility for aflatoxins in baby food (30). Figure 1 illustrates the chromatograms obtained by HPLC-FLD for spiked (A) and non-spiked (B) cereal-based baby food samples. The concentration of aflatoxins in the spiked sample was 0.5 ng/mL for AFB_1_ and AFG_1_ and 0.1 ng/mL for AFB_2_ and AFG_2_.

*AFs occurrence in analyzed samples*

Forty different samples of cereal-based baby foods from available commercial brands in Iran market in two cold (20 samples) and warm (20 samples) seasons were analyzed for AFs (B_1_, B_2_, G_1_, and G_2_) contamination (Table 1). As Table 1 shows, the most frequent occurrence in the cold season was observed for AFB_2,_ which was detected in 60% of assessed samples, followed by AFB_1_ (50%), while AFG_1_ and AFG_2_ were detected only in one (5%) and 4 samples (20%), respectively. On the other hand, the samples from the warm season showed the aflatoxins occurrence for both AFB_1 _and AFB_2_ were 60% (detected in 12 samples) and for AFG_2_ was 30% (detected in 6 samples); while AFG_1_ was not detected in any of the assessed samples. Only two samples taken from the cold season (0.51 ng/g and 0.84 ng/g) exceeded the maximum limit of AFB_1_ (0.5 ng/g) established by the National Standard Organization (15) of Iran; however, six of them (30%) had a concentration beyond European Commission (EU) maximum level of 0.1 ng/g for AFB_1_ (14), with contamination levels of 0.11, 0.14, 0.28, 0.5, 0.51, and 0.84 ng/g. Similarly, from the warm season samples, only one sample exceeded the AFB_1_ level established by the National Standard Organization of Iran, while seven; samples exceeded the European Commission maximum level for AFB_1_. 

There are several studies associated with the presence of aflatoxins in cereals and cereal-based products; for example, Zhang K, Flannery BM, Oles CJ and Adeuya A (31) analyzed a total of 215 retail samples of commercial infant/toddler foods (cereals and teething biscuits) and breakfast cereals collected from three geographical locations in USA for aflatoxins contamination but did not detect any amounts of aflatoxins including B_1_, B_2_, G_1_ and G_2_ in any of the 215 analyzed samples. In another work, Ul Hassan Z, Al Thani R, A. Atia F, Al Meer S, Migheli Q and Jaoua S (32) reported the presence of AFB_1_ in 22% of 67 commercial formula milk and cereal-based baby food samples on the Qatar market which 14-43% of these positive samples had levels higher than the EU maximum limits, while none of B_2_, G_1_, and G_2_ were found at detectable levels in any of the assessed samples. In a similar study to our work, 18 samples out of 26 (69%) breakfast cereal samples purchased from supermarkets in Lisbon (Portugal) were contaminated with AFB_1_, 7 samples (27%) contaminated with AFB_2_ and one sample was contaminated with AFG_1_, while AFG_2_ was not detected in any of the analysed samples (33). The levels of AFB_1_, as the most carcinogenic aflatoxin, in the current study were also comparable to those reported by Ibáñez-Vea M, Martínez R, González-Peñas E, Lizarraga E and de Cerain AL (34) in breakfast cereal samples from the Spanish market (0.051–0.130 µg/kg), Villa P and Markaki P (35) (0.05–4.3 µg/kg of AFB_1_, with an incidence of 56.3%) in samples from Greece and Iqbal SZ, Rabbani T, Asi MR and Jinap S (36) (0.04–6.9 µg/kg of AFB_1_, with an incidence of 41%) in samples from the Pakistan markets. Both similarities and differences between the results of our study and the mentioned studies above could be due to a number of factors, including the differences in analytical methods, the source of cereal ingredients, geographical regions, climatic factors, seasonal variability, and product brands sampled. It is important to note that statistical comparison between AF levels in cold season and warm season were not remarkable (*p* > 0.05). The results were similar to a research caried out by Elaridi et al. who studied concentration of aflatoxin M_1_ and ochratoxin in cereal-based baby food samples in Lebanon. Similarly, the sampling was done in two different seasons and reported no significant difference between samples from different seasons (37).

*Estimation of food consumption*

In this study, the mean consumption rate of cereal-based baby food in babies aged up to 24 months (in three groups including 6-12 months, 12-18 months, and 18-24 months) was assumed to be 100 g/day based on recommended serving size for each brand by producers and using scientific literature reports about the average amounts of cereal-based baby food daily consumption (23). This amount was then used to calculate the estimated dietary exposure and subsequently estimate the risk assessment of consumption of cereal-based baby foods in studied age groups in this current work. In order to determine the mean consumption rate, some information was taken from a survey by the National Nutrition & Food Technology Research Institute of Iran (NNFTRI) (38); accordingly: 75.92% of babies in Iran start complementary food by solid, semi-solid, and hard complementary foods from ages ranged 6-8 months; 93.2% babies < 24 months were fed by complementary foods which 88.8% of them had the minimum meal frequency, and 84.4% of them had an acceptable variety in their daily complementary food. According the used questionnaire in NNFTRI survey 77.8% of babies consumed cereal-based complementary food the day before fill out the questionnaire, indicating the domination of the cereal-based foods as complementary food in the ages lower than 24 months in Iran. 

*Estimation of AFB*_1_ *and total AFs exposures*

**Figure 2:**
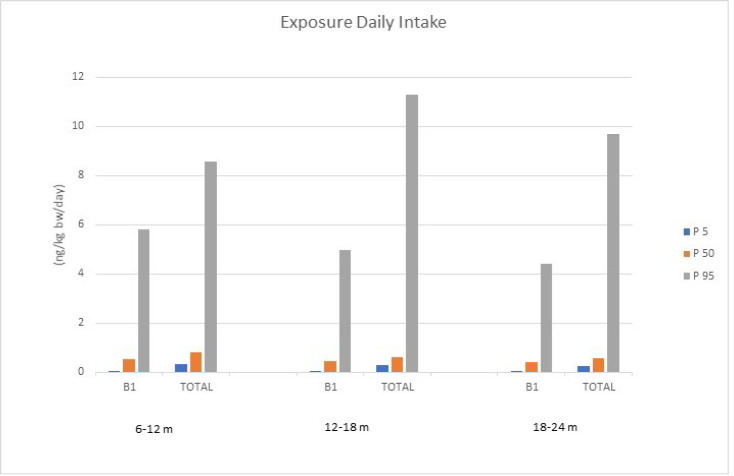
Aflatoxin B_1_ and total aflatoxins Daily Exposure (ng/kg bw/day) in different percentiles and age groups for cereal-based baby food consumption

In general, the deterministic (point estimate procedure), semi-probabilistic method, and probabilistic modelling (Monte Carlo simulation) have been used to evaluate dietary exposure to hazardous compounds in foodstuff. The Monte Carlo simulation is a computational system for stochastic modelling, which has been considered the most promising method with the capability to make risk predictions and delete uncertainties (17). Consequently, the Monte Carlo simulation approach was used in order to estimate dietary exposure of three different age groups, including 6-12 months, 12-18 months, and 18-24 months to AFB_1_ and total AFs (TAFs; the sum of B_1_, B_2_, G_1_, and G_2_ aflatoxins). As is presented in Table 2, the calculations were performed for 5, 50, and, 95 percentiles and the simulation approach forecasted results as distribution. The Monte Carlo simulation model showed significant differences among different age groups with the highest estimated dietary exposure to both AFB_1_ and TAFs for 6-12 months aged babies, representing 5.81 ng/kg BW/day and 8.55 ng/kg BW/day regardless of the percentile, respectively. This can be explained by the lower body weight of babies in this group, as dietary exposure was obtained by multiplying the aflatoxins concentrations in the amount of consumption divided by body weight. Hence, lower body weight reflects a higher dietary exposure. Considering the results of the estimated dietary exposure using the Monte Carlo simulation approach for percentile 50 as the median of population, the lowest dietary exposure was recorded in 18-24 months age group for AFB_1_, and the highest dietary exposure for 6-12 months for TAFs, representing 0.41 ng/kg BW/day and 0.82 ng/kg BW/day, respectively. As is shown in Table 2, estimated dietary exposure results indicate a remarkable difference among the same age group for different percentiles; for instance, in the 6-12 months age group, 95% of the population (P5) had an estimated dietary exposure lower than 0.05 ng/kg BW/day to AFB1 indicating relatively low-risk exposure for this population, while 5% of this group (P95) was exposed to 5.81 ng/kg BW/day to TAFs, which is nearly 10-fold of estimated dietary exposure for the median of the studied population (P50). In the 6-12 months age group, for example, estimated dietary exposure to TAFs for percentile 5 (P5) was lower than 0.32 ng/kg BW/day, which is about one-third of the estimated dietary exposure for the median of population, while for 95 percentile this amount was quite high (8.55 ng/kg BW/day). For the other two age groups, 12-18 months and 18-24 months, 5% of the population (P95) estimated dietary exposure was relatively high compared to the median of the studied population (P50); this amount for the 12-18 months age group was 4.96 for AFB_1_ and 11.30 ng/kg BW/day for TAFs; and for 18-24 months the aged group was 4.39 for AFB_1_ and 9.69 ng/kg BW/day for TAFs, respectively; representing approximately a 10-fold of the median of these populations in the case of AFB_1_. Figure 2 depicts exposures to AFB1 and total AF in different groups and percentiles. However, as AFB_1_ is classified as the Group 1 of carcinogens to humans by the IARC, any threshold of dietary exposure for this toxin has not been established (6).

On the other hand, as aflatoxins may be widespread in many foodstuffs, achievement of “zero” dietary exposure seems to be impossible; therefore, it is desirable to reduce dietary exposure to aflatoxins to as low as possibly achievable (39). As the IARC (2015) has reported that long-term dietary exposure to aflatoxins leads to several health problems such as impaired or stunted growth in children, hepatocellular carcinoma (liver cancer), and long-lasting health complaints later in life (40). Therefore, it is vital that an assessment of dietary exposure to AFs in foodstuffs is conducted to show the current situation and determine a mitigation strategy to inform legislation policy to regulate the levels of aflatoxins in food. In this context, several countries have conducted research and have reported dietary exposure to aflatoxins through the consumption of cereal-based baby foods, which can be affected by either consuming a lot of moderately contaminated foods or eating a moderate amount of highly contaminated foods. In accordance with our results, Herrera M, Bervis N, Carramiñana JJ, Juan T, Herrera A, Ariño A and Lorán S (39) reported an estimated daily intake of AFB_1_ ranging from 0.17 to 0.37 ng/kg BW/day in cereal-based baby foods collected from a random sample of supermarkets, pharmacies and organic food retailers in the Cantabria and Aragón regions of Spain. Similarly, Bakker G, Sizoo E, Jekel A, Pereboom-De Fauw D, Schothorst R and Van Egmond H (41) reported an estimated daily intake of 0.42 ng AFB_1_/kg BW/day for children between 2–6 years old in the Netherlands. Cano-Sancho G, Sanchis V, Marín S and Ramos A (42) also stated that breakfast cereals are the main contributor to the total aflatoxin dietary intake for children with an estimated daily intake of 0.106 ± 0.113 ng/kg BW/day. 

In another study, Ojuri OT, Ezekiel CN, Eskola MK, Šarkanj B, Babalola AD, Sulyok M, Hajšlová J, Elliott CT and Krska R (43) reported a dietary exposure of 5.5-51192 ng AFB_1_/kg BW/day (Median 528) from Tom bran (which is usually formulated from several whole grains including maize, peanuts, wheat, soybean and millet), 5.7-3211 ng AFB_1_/kg BW/day (median 20) from Ogi (a maize-based fermented gruel), 3.5-426 ng AFB_1_/kg BW/day (median 7) from infant formula (included products with a mix of milk and cereal (*e.g*., maize, oats, rice or wheat depending on the brand(, and 2.5 -639 ng AFB_1_/kg BW/day (median 91) from family cereal (a maize product) for infants and young children in Nigeria. They also reported a dietary exposure of 40.5-54892 ng/kg BW/day (median 641), 41.8-3539 ng/kg BW/day (median 68), 25.7-533 ng/kg BW/day (median 55), and 27-902 ng/kg BW/day (median 179) to TAFs through Tom bran, Ogi, infant formula, and family cereal diet, respectively. Similar to our results, the exposures varied through age groups; i.e., the mean exposure to AFB_1_ for 12–24 months age group (2985 ng/kg BW/day) was significantly higher than those for <12 months age group (282 ng/kg BW/day) as well as to the sum of aflatoxins the dietary exposure for 12–24 months age group and for under 12 months age group were 3840 ng/kg BW/day and 387 ng/kg BW/day, respectively. This observation demonstrates the correlation between the age of the child and the exposure increase, in that as a child grows up, its diet changes from mother’s breast milk to baby formula and breakfast cereals which are highly prone to mycotoxin contamination (44).

*Risk characterization*

*The margin of Exposure estimation*

Due to geno-toxicity and carcinogenicity of aflatoxins, the Joint FAO/WHO Expert Committee on Food Additives (JECFA) has not set a tolerable daily intake for them; hence, the risk characterization for aflatoxins is based on margins of exposure (MoE) (45, 46). Indeed, MoE is a ratio between the toxicity effects and dose of a hazardous compound which causes a low but measurable response (benchmark dose, BMD) and the estimated dietary exposure (23). In general, the benchmark dose lower confidence limit of 10% (BMDL_10_) has been suggested in order to calculate MoE, especially in the case of aflatoxins, which is an estimation of the lowest dose that is 95% certain to cause no more than 10% cancer incidence (47). According to the EFSA scientific committee guidance, MoE values equal to or higher than 10,000, based on the BMDL_10_ from an animal study, are considered a cut-off point of low concern from a public health point of view and can be assumed as a low priority for risk management actions (25). However, the output results from the Monte Carlo simulation method showed, except about percentile 95 for AFB_1_ in 18-24 months age group, an estimated MoE lower than 10,000 for the all percentiles (5, 50, and 95 percentiles) for all age groups calculated in this study to both AFB_1_ and TAFs, indicating a health risk from AFB_1_ and TAFs exposure through cereal-based baby food consumption in less than 24 months age population. Table 3 shows the forecasted distribution for MoE of aflatoxin B1 and total aflatoxin obtained from the Monte Carlo simulation. The 5th percentile, as the worst scenario (those who are at high exposure), represented a MoE of 695, 915, and 905 for AFB_1_ in 6-12, 12-18-, and 18-24-months age groups, respectively, and zero for TAFs in all age groups. These results are comparable with those reported by Assunção R, Martins C, Vasco E, Jager A, Oliveira C, Cunha SC, Fernandes JO, Nunes B, Loureiro S and Alvito P (48), who obtained a MoE below 10,000 of AFB_1_ through consumption of breakfast cereals, infant cereals, and biscuits by Portuguese children. A potential health risk increase due to the consumption of breakfast cereals containing aflatoxins, especially in those with a high consuming amount (percentiles 90, 95, and 99) has also been stated by Assuncao R, Vasco E, Nunes B, Loureiro S, Martins C and Alvito P (49); the AFB_1_ was the major contributor for the risk with a total MoE below 10,000. Based on the results of this study, it may be postulated that consumption cereal-based baby food from Iran market could lead to increased health risk in children aged < 24 months, which the higher risk in this age group can be explained by an exceptionally high intake in infants and children in relation to their body weight (50).

*Cancer risk*

According to cancer potency estimation and the prevalence of HBsAg+ in Iran (1.7%) (51) , the results indicate a high cancer risk for high consumers (worst scenario, P95) of cereal-based baby food in all age groups i.e. an estimation of 0.08, 0.07, and 0.06 additional cancer cases per 100,000 in the case of AFB1 exposure and 0.12, 0.16, and 0.14 additional cancer cases per 100,000 in the case TAFs exposure in the 6-12 months, 12-18 months, and 18-24 months age groups, respectively (Table 3), indeed, aflatoxins exposure through cereal-based baby food consumption can roughly add 1.5 new liver cancer patient per 1 million in a year. Given that AFs particularly AFB_1_ are highly carcinogenic and cause hepatocellular carcinoma in humans and it is important to consider immediate intervention (6, 44). 

*Risk ranking*

Recently, a promising method, named risk ranking, has been recommended by the Codex Alimentarius Commission (CAC) (52), which can be used to assess identified hazards; in this case, risk of contaminants in foods evaluate by scoring the probability of risk according to a set of variables. Each variable has a risk category from severe to low, and scores are calculated based on the likely outcome according to frequency data, the contaminants levels, potential repercussions, and the published data in the literature that have described the contaminant.

Risk ranking of a contaminant helps risk managers to set priorities in food safety issues from accepting a risk (if relatively low) through to mitigating risks. Several criteria with their scoring guidance, including toxicities, risk control difficulties, severities of risk, brand reputation in society, a maximum level of detection, and rates of detection, have been established by CAC for risk ranking (Table 4). Accordingly, the scores for a food contaminant can be calculated using formula 3 as described by CAC (52):

Overall score = first index score × (second index score + third score index + fourth score index + fifth score index + sixth score index)

Equation 4.

The overall score of a particular hazard, in this case, food contaminants, calculated by the risk ranking method, is then classified into four categories, including low risk, medium risk, relatively high risk and high risk with overall scores of <50 (low risk); 50-75 (medium risk); 75-100 (relatively high risk); >100 (high risk).

To date, to the best of our knowledge, very little information is available in the literature on using the risk ranking approach mentioned above. However, when applied to the hazard of aflatoxins in cereal-based baby foods, this risk ranking method categorized the presence of AFB_1_ as a high risk for babies that consume cereal-based baby food, as AFB_1_ overall score was 110, which classifies AFB_1_ into high-risk group (Table 5). 

Furthermore, the overall risk ranking score for AFG_1_ was 60, indicating a moderate risk due to the presence of AFG_1_ in cereal-based baby food for babies aged >24 months. Regarding the overall risk scores for AFB_2_ (28) and AFG_2_ (22), these aflatoxins can be classified as low-risk contaminants in cereal-based baby food for babies aged >24 months.

The results of the risk ranking of the AFs when taken together with the estimated dietary exposure, margins of exposure and the potential risk of cancer to AFs through the rates of consumption of cereal-based baby food, indicates that the presence of AFB_1_ in cereal-based baby foods is a serious health risk for babies and demands the attention of risk managers to reduce or eliminate this risk for the most vulnerable sector of society. 

*Management advises*

The infant food producers are advised to put the importance of mycotoxins content in their priorities. Enough information and training must be provided for manufacturers, parents, and health care professionals to reduce the health risk associated with mycotoxins, particularly aflatoxins. It is vital to keep the levels of contaminants low in order to secure public health. Eventually, inspection and surveillance should be constant, extensive, and must be carried out by the government and related ministries because the final product quality depends on accurate control at every step of the manufacturing process.

**Table 1:** Concentration of aflatoxins (ng/g) in cereal-based baby food samples in two difference seasons from different commercial brands in Iran market

**no**	**Cold season samples**				**Warm season samples**			
	**AFB**_1_	**AFB**_2_	**AFG**_1_	**AFG**_2_	**AFB**_1_	**AFB**_2_	**AFG**_1_	**AFG**_2_
1	0.04	0.01	<LOQ	<LOQ	<LOQ	<LOQ	<LOQ	<LOQ
2	0.5	0.04	<LOQ	<LOQ	0.38	0.04	<LOQ	<LOQ
3	0.09	0.01	<LOQ	<LOQ	0.09	0.01	<LOQ	<LOQ
4	<LOQ	<LOQ	<LOQ	<LOQ	<LOQ	<LOQ	<LOQ	0.01
5	<LOQ	<LOQ	<LOQ	<LOQ	0.05	<LOQ	<LOQ	<LOQ
6	0.84	0.08	<LOQ	0.02	0.7	0.08	<LOQ	0.02
7	0.51	0.05	<LOQ	0.02	0.3	0.03	<LOQ	0.01
8	0.14	0.02	<LOQ	0.08	0.15	0.02	<LOQ	0.08
9	<LOQ	<LOQ	<LOQ	0.02	<LOQ	<LOQ	<LOQ	0.02
10	<LOQ	<LOQ	<LOQ	<LOQ	0.15	0.01	<LOQ	0.01
11	<LOQ	<LOQ	<LOQ	<LOQ	<LOQ	<LOQ	<LOQ	<LOQ
12	0.05	0.01	<LOQ	<LOQ	0.17	0.02	<LOQ	<LOQ
13	<LOQ	0.01	<LOQ	<LOQ	0.05	0.01	<LOQ	<LOQ
14	<LOQ	<LOQ	<LOQ	<LOQ	0.04	0.01	<LOQ	<LOQ
15	0.28	0.03	<LOQ	<LOQ	<LOQ	<LOQ	<LOQ	<LOQ
16	<LOQ	<LOQ	<LOQ	<LOQ	<LOQ	<LOQ	<LOQ	<LOQ
17	<LOQ	0.01	<LOQ	<LOQ	0.31	0.03	<LOQ	<LOQ
18	0.04	0.01	<LOQ	<LOQ	<LOQ	<LOQ	<LOQ	<LOQ
19	<LOQ	<LOQ	<LOQ	<LOQ	<LOQ	<LOQ	<LOQ	<LOQ
20	0.11	0.03	<LOQ	<LOQ	0.06	0.02	<LOQ	<LOQ
Mean	0.13	0.0155	<LOQ	0.007	0.1225	0.014	<LOQ	0.0075

**Table 2:** Dietary aflatoxins exposure estimation (ng/kg bw/day) of babies aged 6-24 months by Monte Carlo simulation

**Age groups**	**AFB**_1_			**Total AFs**		
	**P5**	**P50**	**P95**	**P5**	**P50**	**P95**
6-12	0.05	0.54	5.81	0.32	0.82	8.55
12-18	0.04	0.46	4.96	0.27	0.60	11.30
18-24	0.04	0.41	4.39	0.25	0.55	9.69

**Table 3:** The margin of exposure and Cancer potency resulted from AFs exposure from baby foods consumption for 6-24 months old babies simulated by Monte Carlo. (cancer potency value: additional cancer cases per 100,000 people)

**Age groups**	**AFB**_1_								**Total AFs**							
	**P5**			**P50**			**P95**		**P5**			**P50**			**P95**	
	MoE	Cancer potency		MoE	Cancer potency		MoE	Cancer potency	MoE	Cancer potency		MoE	Cancer potency		MoE	Cancer potency
6-12	69.5	0.0007		731.7	0.0008		7814.7	0.08	0	0.0047		588.7	0.012		1208	0.12
12-18	81.5	0.0005		861.7	0.0006		9102.2	0.07	0	0.0040		695.3	0.008		1416.2	0.16
18-24	90.4	0.0005		964.4	0.0006		10351.8	0.06	0	0.0037		778.1	0.008		1581.4	0.14

**Table 4:** Identification of food hazards and risk ranking score evaluation criteria

**Index**	**Index value** **(score = 5)**	**Index value** **(score = 4)**	**Index value** **(score = 3)**	**Index value** **(score = 2)**
Toxicity	High	Relatively high	Medium	Low
Degree of difficulty in risk control	Difficult	Poor	Potentially poor	Capable
Severity	Serious	Relatively serious	Medium	Noteworthy
Social reputation	Serious	Relatively serious	Medium	Noteworthy
Maximum amount of detection residue (μg/kg)	>5000	1000-5000	500-1000	0-500
Detection rate^*^%	>10	8-10	6–8	4–6

**Table 5:** Identification of mycotoxin risk degrees

**Risk factors**	**Toxicity**	**difficulty in risk control**	**Severity**	**Social reputation**	**Detection residue** **(**_μ_ **g/kg)**	**Detection rate (%)**	**Overall score**	**Risk** **degree**
AFB_1_	High (5)	Difficult (5)	Serious (5)	Serious (5)	0-500 (2)	>10 (5)	110	High
AFB_2_	Low (2)	Potentially poor (3)	Medium (3)	Noteworthy (2)	0-500 (2)	>10 (5)	30	Low
AFG_1_	Relatively high (4)	Potentially poor (3)	Relatively serious (4)	Relatively serious (4)	0-500 (2)	0 (2)	60	medium
AFG_2_	Low (2)	Potentially poor (3)	Medium (3)	Noteworthy (2)	0-500 (2)	4-6 (2)	24	low

## Conclusion

Aflatoxins (AFs) occurrence in cereal-based foods is a global health concern as they are used worldwide as complementary foods in babies’ diets. Results showed an occurrence ranging 20% to 60% for B_1_, B_2_, and G_2_ aflatoxins in cereal-based baby foods in Iran, while AFG_1_ was not detected. 10% and 30% of total samples were exceeded the maximum limit of AFB_1_ (0.5 ng/g) established by National Standard Organization of Iran and European Commission maximum (0.1 ng/g), respectively. The Monte Carlo simulation model showed a significant difference in estimated dietary exposure among three assessed age groups (6-12 months, 12-18 months, and 18-24 months) with the highest estimated dietary exposure to both AFB_1_ and total AFs for 6-12 months aged babies. In general, except in one case, a MoE lower than 10,000 was estimated by Monte Carlo simulation approach for all 5, 50, and 95 percentiles in all the age groups to AFB_1_ and total AFs, indicating a health concern about AFB_1_ and total AFs exposure through cereal-based baby food consumption from Iran in children aged <24 months. For the AFB_1_ and TAFs analyzed, a high cancer risk for high consumers (P95) of cereal-based baby food aged <24 months was also estimated, calling for immediate intervention due to serious claims that AFB_1_ is highly carcinogenic, inducing hepatocellular carcinoma. According to the risk ranking results, the presence of AFB_1_ is a high risk for babies who consume cereal-based baby food, which demands the attention of risk managers to reduce or eliminate this risk for the most vulnerable sector of society; however, the presence of AFG_1_, AFB_2_ and AFG_2_ were classified as moderate risk, low risk, low risk, respectively, in cereal-based baby foods for baby consumers whose aged <24 months.

## References

[1] Majeed M, Khaneghah AM, Kadmi Y, Khan MU, Shariati MA (2018). Assessment of ochratoxin A in commercial corn and wheat products. Curr. Nutr. Food. Sci..

[2] Nejad ASM, Heshmati A, Ghiasvand T (2019). The occurrence and risk assessment of exposure to aflatoxin M1 in ultra-high temperature and pasteurized milk in Hamadan province of Iran. Osong. Public Health Res. Perspect..

[3] Khaneghah AM, Fakhri Y, Gahruie HH, Niakousari M, Sant’Ana AS (2019). Mycotoxins in cereal-based products during 24 years (1983–2017): A global systematic review. Trends Food Sci. Technol..

[4] Singh J, Mehta A (2020). Rapid and sensitive detection of mycotoxins by advanced and emerging analytical methods: A review. Food Sci. Nutr..

[5] Kamkar A, Fallah AA, Mozaffari Nejad AS (2014). The review of aflatoxin M1 contamination in milk and dairy products produced in Iran. Toxin Rev..

[6] Cancer IAfRo (1993). Monograph on the Evaluation of Carcinogenic Risk to Humans: Some Naturally Occurring Substances, Some Foodstuffs and Constituents, Heterocyclic Aromatic Amines and Mycotoxins. IARC Monograph, France.

[7] Raiola A, Tenore GC, Manyes L, Meca G, Ritieni A (2015). Risk analysis of main mycotoxins occurring in food for children: An overview. Food Chem. Toxicol..

[8] Eslami M, Mashak Z, Heshmati A, Shokrzadeh M, Mozaffari Nejad AS (2015). Determination of aflatoxin B1 levels in Iranian rice by ELISA method. Toxin Rev..

[9] Alamu EO, Gondwe T, Akello J, Sakala N, Munthali G, Mukanga M, Maziya-Dixon B (2018). Nutrient and aflatoxin contents of traditional complementary foods consumed by children of 6–24 months. Food Sci. Nutr..

[10] Manova R, Mladenova R (2009). Incidence of zearalenone and fumonisins in Bulgarian cereal production. Food Control..

[11] Boon P, Bakker M, Van Klaveren J, Van Rossum C (2013). Risk assessment of the dietary exposure to contaminants and pesticide residues in young children in the Netherlands. European J. Nutr.Food Saf..

[12] Scheuplein R, Charnley G, Dourson M (2002). Differential sensitivity of children and adults to chemical toxicity. I. Biological basis. Regul. Toxicol. Pharmacol..

[13] Huybrechts I, Sioen I, Boon PE, Ruprich J, Lafay L, Turrini A, Amiano P, Hirvonen T, De Neve M, Arcella D, Moschandreas J, Westerlund A, Ribas-Barba L, Hilbig A, Papoutsou S, Christensen T, Oltarzewski M, Virtanen S, Rehurkova I, Azpiri M, Sette S, Kersting M, Walkiewicz A, Serra-Majem L, Volatier JL, Trolle E, Tornaritis M, Busk L, Kafatos A, Fabiansson S, De Henauw S, Van Klaveren JD (2011). Dietary exposure assessments for children in Europe (the EXPOCHI project): rationale, methods and design. Arch. Public Health.

[14] Ireland FSAo (2009). Mycotoxins in Food. Toxicology Factsheet Series.

[15] (2020). (ISIRI), Standard number 5925, Maximum tolerated levels of mycotoxins in food and feeds.

[16] Lorán S, Bayarri S, Conchello P, Herrera A (2010). Risk assessment of PCDD/PCDFs and indicator PCBs contamination in Spanish commercial baby food. Food Chem. Toxicol..

[17] Nematollahi A, Kamankesh M, Hosseini H, Ghasemi J, Hosseini-Esfahani F, Mohammadi A, Mousavi Khaneghah A (2020). Acrylamide content of collected food products from Tehran’s market: A risk assessment study. Environ. Sci. Pollut. Res..

[18] Taghizadeh SF, Rezaee R, Badibostan H, Karimi G (2020). Aflatoxin B1 in walnuts: A probabilistic cancer risk assessment for Iranians. Toxicol. Environ. Chem..

[19] Alvito PC, Sizoo EA, Almeida CM, van Egmond HP (2010). Occurrence of aflatoxins and ochratoxin A in baby foods in Portugal. Food Anal. Methods.

[20] ISIRI (Institute of Standard and Industrial Research of I R Iran) (2012). Determination content of Aflatoxins cleaned up by immunoaffinity column with high performance liquid chromatography in food and feed. National Standard No. 6872.

[21] Bashiry M, Mohammadi A, Hosseini H, Kamankesh M, Aeenehvand S, Mohammadi Z (2016). Application and optimization of microwave-assisted extraction and dispersive liquid–liquid microextraction followed by high-performance liquid chromatography for sensitive determination of polyamines in turkey breast meat samples. Food Chem..

[22] Nejad AS, Heshmati A, Ghiasvand T (2020). The occurrence and risk assessment of aflatoxin M1 in Cheeses samples from Hamadan, Iran. Iran. J. Pharm. Sci..

[23] Badibostan H, Feizy J, Daraei B, Shoeibi S, Rajabnejad SH, Asili J, Taghizadeh SF, Giesy JP, Karimi G (2019). Polycyclic aromatic hydrocarbons in infant formulae, follow-on formulae, and baby foods in Iran: An assessment of risk. Food Chem. Toxicol..

[24] WHO Multicentre Growth Reference Study Group (2006). WHO child growth standards based on length/height, weight and age. Acta Paediatr. Suppl..

[25] Schrenk D, Bignami M, Bodin L, Chipman JK, Del Mazo J, Grasl-Kraupp B, Hogstrand C, Hoogenboom LR, Leblanc JC, Nebbia CS, Nielsen E, Ntzani E, Petersen A, Sand S, Schwerdtle T, Vleminckx C, Marko D, Oswald IP, Piersma A, Routledge M, Schlatter J, Baert K, Gergelova P, Wallace H (2020). Risk assessment of aflatoxins in food. EFSA. J..

[26] Benford D, Bolger PM, Carthew P, Coulet M, DiNovi M, Leblanc JC, Renwick AG, Setzer W, Schlatter J, Smith B, Slob W, Williams G, Tanja Wildemann, Wildemann T (2010). Application of the margin of exposure (MOE) approach to substances in food that are genotoxic and carcinogenic. Food Chem. Toxicol..

[27] Heshmati A, Mozaffari Nejad ASM, Ghyasvand T (2020). The occurrence and risk assessment of aflatoxin M1 in yoghurt samples from Hamadan, Iran. Open Public Health J..

[28] Rambla-Alegre M, Esteve-Romero J, Carda-Broch S (2012). Is it really necessary to validate an analytical method or not? That is the question. J. Chromatogr. A.

[29] European Commission D (2017). Guidance document on analytical quality control and method validation procedures for pesticides residues analysis in food and feed.

[30] Commission E (2006). Commission Regulation (EC) No 1881/2006 of 19 December 2006 setting maximum levels for certain contaminants in foodstuffs. Off. J. Eur. Union..

[31] Zhang K, Flannery BM, Oles CJ, Adeuya A (2018). Mycotoxins in infant/toddler foods and breakfast cereals in the US retail market. Food Addit. Contam..

[32] Ul Hassan Z, Al Thani R A (2018). Co-occurrence of mycotoxins in commercial formula milk and cereal-based baby food on the Qatar market. Food Addit. Contam..

[33] Martins C, Assunção R, Cunha SC, Fernandes JO, Jager A, Petta T, Oliveira CA, Alvito P (2018). Assessment of multiple mycotoxins in breakfast cereals available in the Portuguese market. Food Chem..

[34] Ibáñez-Vea M, Martínez R, González-Peñas E, Lizarraga E, de Cerain AL (2011). Co-occurrence of aflatoxins, ochratoxin A and zearalenone in breakfast cereals from Spanish market. Food Control..

[35] Villa P, Markaki P (2009). Aflatoxin B1 and ochratoxin A in breakfast cereals from Athens market: Occurrence and risk assessment. Food Control..

[36] Iqbal SZ, Rabbani T, Asi MR, Jinap S (2014). Assessment of aflatoxins, ochratoxin A and zearalenone in breakfast cereals. Food Chem..

[37] Elaridi J, Dimassi H, Hassan H (2019). Aflatoxin M1 and ochratoxin A in baby formulae marketed in Lebanon: Occurrence and safety evaluation. Food Control..

[38] Abdollahi M (2018). Determination of anthropometric status, nutritional indicators and growth and development, and some indicators for evaluating health system services in children under 5 years old in Iran. NNFTRI..

[39] Herrera M, Bervis N, Carramiñana JJ, Juan T, Herrera A, Ariño A, Lorán S (2019). Occurrence and exposure assessment of aflatoxins and deoxynivalenol in cereal-based baby foods for infants. Toxins.

[40] Wild CP, Miller JD, Groopman JD (2015). Mycotoxin Control in Low-and Middle-income Countries. International Agency for Research on Cancer, France.

[41] Bakker G, Sizoo E, Jekel A, Pereboom-De Fauw D, Schothorst R, Van Egmond H (2009). Determination of mean daily intakes of aflatoxin B1, aflatoxin M1, ochratoxin A, trichothecenes and fumonisins in 24-hour diets of children in the Netherlands. World Mycotoxin J..

[42] Cano-Sancho G, Sanchis V, Marín S, Ramos A (2013). Occurrence and exposure assessment of aflatoxins in Catalonia (Spain). Food Chem. Toxicol..

[43] Ojuri OT, Ezekiel CN, Eskola MK, Šarkanj B, Babalola AD, Sulyok M, Hajšlová J, Elliott CT, Krska R (2019). Mycotoxin co-exposures in infants and young children consuming household-and industrially-processed complementary foods in Nigeria and risk management advice. Food Control..

[44] Gong YY, Watson S, Routledge MN (2016). Aflatoxin exposure and associated human health effects, a review of epidemiological studies. J. Food Saf..

[45] Benford D, Bolger PM, Carthew P, Coulet M, DiNovi M, Leblanc JC, Renwick AG, Setzer W, Schlatter J, Smith B, Slob W, Williams G, Wildemann T (2010). Application of the margin of exposure (MOE) approach to substances in food that are genotoxic and carcinogenic. Food Chem. Toxicol..

[46] Kamala A, Kimanya M, Lachat C, Jacxsens L, Haesaert G, Kolsteren P, Ortiz J, Tiisekwa B, De Meulenaer B (2017). Risk of exposure to multiple mycotoxins from maize-based complementary foods in Tanzania. J. Agric. Food Chem..

[47] Moghaddam AF, Rychlik M, Hosseini H, Janat B, Yazdanpanah H, AliAbadi M (2019). Risk associated with the intake of aflatoxin M1 from milk in Iran. World Mycotoxin J..

[48] Assunção R, Martins C, Vasco E, Jager A, Oliveira C, Cunha SC, Fernandes JO, Nunes B, Loureiro S, Alvito P (2018). Portuguese children dietary exposure to multiple mycotoxins–an overview of risk assessment under MYCOMIX project. Food Chem. Toxicol..

[49] Assuncao R, Vasco E, Nunes B, Loureiro S, Martins C, Alvito P (2015). Single-compound and cumulative risk assessment of mycotoxins present in breakfast cereals consumed by children from Lisbon region, Portugal. Food Chem. Toxicol..

[50] Serrano A, Font G, Ruiz M, Ferrer E (2012). Co-occurrence and risk assessment of mycotoxins in food and diet from mediterranean area. Food Chem..

[51] Razavi-Shearer D, Gamkrelidze I, Nguyen MH, Chen DS, Van Damme P, Abbas Z, Abdulla M, Abou Rached A, Adda D, Aho I, Akarca U (2018). Global prevalence, treatment, and prevention of hepatitis B virus infection in 2016: a modelling study. The lancet Gastroenterology & hepatology..

[52] Li P, Ding X, Bai Y, Wu L, Yue X, Zhang L, Svalova VA (2018). Risk assessment. Risk Assessment and Prediction of Aflatoxin in Agro-Products.

